# Habitat and spatial trends of U.K. wintering waterbird populations over 50 years

**DOI:** 10.1111/cobi.70310

**Published:** 2026-05-14

**Authors:** Blaise Martay, Philipp H. Boersch‐Supan, James W. Pearce‐Higgins, Graham E. Austin, Niall H. K. Burton, David Noble, Kirsi Peck, Simon R. Wotton, Teresa M. Frost

**Affiliations:** ^1^ British Trust for Ornithology Thetford UK; ^2^ Conservation Science Group, Department of Zoology University of Cambridge Cambridge UK; ^3^ Joint Nature Conservation Committee Peterborough UK; ^4^ RSPB Centre for Conservation Science Royal Society for the Protection of Birds Sandy UK

**Keywords:** climate change, habitat, migration, phenology, populations, waterbirds, Aves acuáticas, cambio climático, fenología, hábitat, migración, poblaciones, 水鸟, 种群, 迁徙, 气候变化, 栖息地, 物候

## Abstract

The United Kingdom is an important wintering ground for millions of waterbirds. Most U.K. wintering waterbird populations increased between 1970 and the mid‐1990s, but declined thereafter. We examined U.K. population indices in 46 wintering waterbird species in two 25‐year periods, 1970–1994 and 1995–2019, to identify which waterbird groups were vulnerable to declines. We modeled abundance to examine whether spatial patterns in population change were consistent with changes in habitat quality, climate, or phenology. We used the resulting model outputs to test whether there were patterns in these relationships across all monitored wintering waterbirds and within groups based on ecology, migratory strategy, and breeding location. Waterbird populations in the United Kingdom increased from 1970 to 1994, particularly in estuaries, but then showed mixed trends from 1995 to 2019: geese and swan populations increased, and waders declined. Wader declines were least apparent, although still declining, in estuaries and most apparent in reservoirs. Increased breeding season temperatures were linked to population increases for most species in both periods, although there was no beneficial impact of climate warming for migrants from 1995 to 2019. We found no evidence that apparent declines were due to phenological changes. These results highlight the need for more research into why U.K. wintering waterbird trends have become more negative over the past 25 years.

## Abstract (es)

Tendencias del hábitat y espaciales de las poblaciones de aves acuáticas invernantes del Reino Unido a lo largo de 50 años

**Resumen:**

El Reino Unido es una importante zona para millones de aves acuáticas durante el invierno. La mayoría de las poblaciones de aves acuáticas invernantes del Reino Unido aumentaron entre 1970 y mediados de la década de 1990, pero disminuyeron a partir de entonces. Examinamos los índices de población del Reino Unido de 46 especies de aves acuáticas invernantes en dos periodos de 25 años, 1970‐1994 y 1995‐2019, para identificar qué grupos de aves acuáticas eran vulnerables a las disminuciones. Modelamos la abundancia para examinar si los patrones espaciales en el cambio de población eran coherentes con los cambios en la calidad del hábitat, el clima o la fenología. Utilizamos los resultados del modelo para comprobar si existían patrones en estas relaciones entre todas las aves acuáticas invernantes monitorizadas y dentro de los grupos en función de la ecología, la estrategia migratoria y el lugar de cría. Las poblaciones de aves acuáticas en el Reino Unido aumentaron entre 1970 y 1994, especialmente en los estuarios, pero luego mostraron tendencias mixtas entre 1995 y 2019: las poblaciones de gansos y cisnes aumentaron, mientras que las de aves zancudas disminuyeron. La disminución de las aves zancudas fue menos evidente, aunque siguió disminuyendo, en los estuarios, y más evidente en los embalses. El aumento de las temperaturas durante la temporada de cría se relacionó con el incremento de las poblaciones de la mayoría de las especies en ambos periodos, aunque no se observó ningún impacto benéfico del calentamiento climático para las aves migratorias entre 1995 y 2019. No se encontraron indicios de que las disminuciones observadas se debieran a cambios fenológicos. Estos resultados ponen de relieve la necesidad de investigar más a fondo por qué las tendencias de las aves acuáti**ca**s invernantes del Reino Unido se han vuelto más negativas en los últimos 25 años.

## 过去50年间英国越冬水鸟种群的栖息地与空间变化趋势

**【摘要】**英国是数百万水鸟的重要越冬地。1970年至1990年代中期, 英国大部分越冬水鸟种群数量有所增长, 但此后开始下降。本研究分析了1970–1994年和1995–2019年这两个25年周期内, 英国46种越冬水鸟的种群指数, 以确定哪些水鸟类群容易发生种群数量下降。我们建立了种群丰度模型, 以探究种群变化的空间格局是否与栖息地质量、气候或物候学的变化相一致。接下来, 我们利用模型输出结果, 检验了这些关系是否在所有受监测的越冬水鸟中以及在基于生态学、迁徙策略和繁殖地划分的各分组内存在一定规律。结果显示, 1970年至1994年间, 英国水鸟种群数量有所增加, 特别是在河口地区, 但1995年至2019年间则呈现出混合趋势:鹅和天鹅种群数量增加, 而涉禽种群数量减少。涉禽种群数量的下降趋势在河口地区虽存在但最不明显, 而在水库中则最为明显。在两个时期内, 繁殖季节气温升高均与大多数物种种群数量增长相关联, 但在1995年至2019年间, 气候变暖对迁徙鸟类并未产生积极影响。研究未发现鸟类种群数量的明显下降是由物候变化导致的。以上结果强调了需进一步研究过去25年来英国越冬水鸟种群下降趋势加剧背后的成因。**【翻译:胡怡思;审校:聂永刚】**

## INTRODUCTION

The United Kingdom is an important wintering ground for millions of waterbirds (Frost et al., 2019). Some are resident throughout the year, and others migrate throughout northern Europe and the Arctic to breed in the summer months (Spina et al., 2022). Most U.K. wintering waterbird populations increased rapidly from the 1970s to the mid‐1990s, as summarized in the U.K. wintering waterbird indicator, a composite of species indices (Austin et al., 2023). However, since the mid‐1990s, populations have generally declined (Defra, 2025). This pattern of rapid population increases followed by stabilization or population declines has also been found in Arctic breeding birds (Thaxter et al., 2025), many of which winter in the United Kingdom. Identifying drivers of these declines and which groups of species are most vulnerable is vital for effective conservation.

Drivers of changes in U.K. wintering populations of waterbird species are known to include winter and breeding season climate change (Burton et al., 2020, 2023; Maclean et al., 2008), water quality (Burton et al., 2002; Pringle & Burton, 2017), policies on hunting and the use of lead in hunting and fishing (Taylor & Dodd, 2013; Wood et al., 2019), and wetland habitat degradation, loss, and creation (Mander et al., 2007, 2021). Some of these drivers, such as climate change, may operate across species’ annual cycles and flyways (Burton et al., 2023), whereas others, such as hunting legislation, are U.K. specific.

Many widespread drivers could influence the observed population changes across many species at the same time, including changes in habitat quality such as land use and water quality, climate warming, and shifts in migration phenology, reducing the duration individual birds are present in the United Kingdom. Some of these factors will affect the true abundance of wintering birds in the United Kingdom. Others may affect apparent abundance due to the methods used to calculate population indices. For example, if populations were stable but birds were departing earlier from U.K. wintering grounds, counts would decrease in departure months, causing an apparent population decline. It is likely that these drivers interact and vary between species and groups of species. Here, we examined spatiotemporal patterns in U.K. population trends across 46 wintering waterbird species to identify the extent to which they were consistent with these potential drivers of change.

Change in habitat quality over the past 50 years could be a driver of the recent waterbird decline. These habitat changes could be linked to changes in land use or water quality. U.K. water quality (i.e., increasing oxygen and declining nitrogen and phosphate levels) has generally improved in U.K. rivers and estuaries since the mid‐1990s (DEFRA, 2014; Whelan et al., 2022). In general, this is likely to be beneficial for wintering waterbirds in freshwater (Davies & Sylvester‐Bradley, 1995; Møller & Laursen, 2015; Møller et al., 2015). However, in estuaries and coastal sites, water quality improvements have been observed to lead to declines in some waterbird species due to declines in macrozoobenthic food availability (Burton et al., 2002; Pringle & Burton, 2017). The impacts of changes in water quality, therefore, may be indicated by population indices varying between ecological groups and habitats (Pringle & Burton, 2017). Land use can also affect predator densities and availability of breeding habitat, both of which are closely linked to waterbird productivity and population sizes (Amar et al., 2011; Cook et al., 2021; Woodward et al., 2021). However, variation in habitat trends may also be linked to climate change (e.g., Pearce‐Higgins & Holt, 2013).

Climate change is a possible driver of recent waterbird declines. Much research has found positive associations between breeding season and winter temperature for some waterbird populations, often through increased survival (Cook et al., 2021; Gunnarsson et al., 2017; Pavón‐Jordán et al., 2019; Woodward et al., 2021). However, negative associations between temperature and some waterbird populations have also been found due to the impact of temperature on factors such as food, habitat availability, or predation (Boyd, 2013; Pearce‐Higgins et al., 2010), as well as reductions in food and breeding ground availability (Nagy et al., 2021; Rehfisch & Crick, 2003). Climate warming could have passed temperature thresholds beyond which warming switches from being beneficial for some species to being detrimental (Martay et al., 2023). Identifying which species’ population changes are associated with climate and whether these relationships change before and after the mid‐1990s will help identify whether recent declines in wintering waterbirds may be linked to climate warming.

As well as the direct effects of changes in habitat quality and climate, U.K. wintering waterbird population indices could appear to decline because of changes in habitat use or migration phenology. Changes in habitat quality or climate could lead to waterbird populations changing their use of habitats to those less covered by monitoring, which could lead to lower apparent counts of wintering waterbirds, without reflecting a decline in true population size. Likewise, changes in wintering waterbird migration phenology, such as later arrival and earlier departure, could reduce counts at the start and end of winter, reducing the number of bird‐days (BTO, 2017; Massimino et al., 2021). Changes to migration phenology have been found in wintering waterbirds, with spring departures becoming earlier over time as temperatures increase (Burton et al., 2020).

U.K. wintering waterbird population indices could also decline due to climate‐driven range shifts, without reflecting a negative impact of climate change on flyway populations. Increasing temperatures have been linked to migrating waterbirds short stopping (i.e., reducing wintering migration distances as temperatures increase) to overwinter closer to their breeding grounds (Austin & Rehfisch, 2005; Austin et al., 2000; Maclean et al., 2008; Pavón‐Jordán et al., 2019). Although there is considerable evidence of U.K. wintering waterbird abundance declining in the southwestern United Kingdom due to short stopping (Austin & Rehfisch, 2005; Maclean et al., 2008; Pavón‐Jordán et al., 2019), there is little evidence of waterbirds short stopping to the United Kingdom from warmer European wintering grounds (Nightingale, 2023; Woodward et al., 2024). Thus, a decline in U.K. migratory wintering waterbirds could reflect distributional shifts across Europe rather than global population declines. In the United Kingdom and across northwestern Europe, waders and wildfowl have shown long‐term northward or eastward shifts in their wintering distributions, reflecting the breeding origins of most species and warming temperatures (Austin & Rehfisch, 2005; Maclean et al., 2008; Pavón‐Jordán et al., 2019).

We explored recent U.K. wintering waterbird population declines by using site‐level counts to compare patterns of abundance and trends in two 25‐year periods: the period when population indices were generally positive (1970–1994), with the patterns of trends during a period of decline (1995–2019). In both periods, we identified whether population trends varied across different habitats, varied spatially, correlated with temperature (which could indicate potential impacts of climate warming), and showed evidence of being influenced by changes in migration phenology. We examined how these patterns differed between species based on their taxonomy, diet, migratory strategy, and breeding locations. In combination, these analyses were designed to identify which groups of wintering waterbirds changed in abundance most in the United Kingdom and which of the potential hypotheses the observed changes were most consistent with to inform future studies and potential conservation responses.

## METHODS

### Bird data

The U.K. wintering waterbird species indices were generally based on the BTO/RSPB/JNCC Wetland Bird Survey Core Count scheme for most waterbirds, except some geese and swans, for which census data were available. The Wetland Bird Survey (WeBS) was the U.K.’s most comprehensive scheme for monitoring waterbirds. Wildfowl counts began in 1947, and the Birds of Estuaries Enquiry counts began in the late 1960s to cover a wider range of species (Cranswick et al., 1997). These surveys were combined in 1993 to create WeBS (Cranswick et al., 1997). Bird counts on 2066 predefined wetland sites (in the dataset excluding years out of our study period) were carried out by volunteers, ideally on a monthly basis and on a specific date (Austin et al., 2023; BTO, 2017). On average, between 1970 and 2019, 705 sites per year were monitored. If a recorder could not monitor their entire site in a visit, the count was still recorded but flagged as incomplete. Coverage was highest between September and March. Larger sites were generally subdivided into smaller sectors to allow a team to cover the whole site. A large proportion of U.K. estuarine sites and larger lowland inland still waters were monitored, but on other wetland habitats, such as rivers and rocky and open coasts, there were many more unmonitored sites (Méndez et al., 2015).

We used site‐level WeBS count data for 46 species or subspecies for which species detection and coverage were considered robust enough for WeBS indices to be created and reported (Austin et al., 2023). We classified each species by ecology, migratory strategy, and breeding location (Appendix S1). The ecological groups based on taxonomy and ecology were geese and swans (10 species), dabbling ducks and rails (10 species), diving ducks (five species), piscivores (six species), and waders (15 species). The three migratory strategies were residents (17 species), partial migrants (14 species), and migrants (15 species) (Wernham et al., 2002). We classified species by breeding location, identifying where most individuals wintering in the United Kingdom bred for each species. We divided locations into three categories: the United Kingdom (17 species), northwest (i.e., Greenland, North America, and Iceland) (10 species), and northeast (i.e., Scandinavia, northwestern Europe, and Siberia) (13 species). We added a fourth category, mixed breeding locations, for dabbling ducks and rails (six species in total) because all these species breed across large areas of Europe and North America (Wernham et al., 2002) (Appendix S1).

### Climatic and weather data

Wintering waterbird abundance could potentially be influenced by winter and breeding season weather. To avoid assumptions over which weather variables influenced wintering waterbird populations most and to avoid including correlating variables, we carried out exploratory modeling to identify which winter and breeding season weather variables correlated most closely with wintering waterbird populations and included the two selected variables in the larger models described in the “Species‐specific models” section. To carry out the exploratory modeling, we examined the correlations between counts of each of the 46 species in this study and weather variables over the winter months of the WeBS survey (September–March to cover all monitored months and November–February to cover the key wintering period) and during the breeding season prior to the winter monitoring (April–August) known to affect U.K. or northern European wintering waterbird populations or distributions. Weather variables included the North Atlantic Oscillation (NAO) index (winter impact [Pavón‐Jordán et al., 2019]; breeding season [Boyd, 2013]) and U.K. temperature (winter [Burton et al., 2020]; breeding season [Doyle et al., 2020]). The methods and results of this exploratory modeling are in Appendix S2. The NAO index represented the large‐scale climatic conditions for the North Atlantic Ocean; strong positive values were associated with above‐normal temperature and rainfall over northern Europe and Scandinavia (Hurrell, 1995). This index was downloaded from Hurrell et al. (2020). The U.K. temperature data were taken from the mean monthly U.K. temperatures derived from 1‐km gridded temperatures (Hollis et al., 2019; Met Office, 2018). The exploratory modeling showed that the winter weather variable that best explained the variation in abundance was the U.K. winter temperature from September to March (U.K. September–March winter temperature explained more population abundance variation than other winter variables for 80% of species). The breeding season weather variable that best explained the variation in abundance was U.K. breeding season temperature (U.K. breeding season temperature explained more population abundance variation than breeding season NAO for 91% of species) (Appendix S2).

### Habitat data

The WeBS database classifies sites into six habitat categories: open coast, estuarine, river or marsh, natural inland still water, gravel pit, and reservoir. For each species, we examined the number of counts within each habitat. If fewer than 50 counts were present in a habitat across the period in question, we removed data from that habitat for the species. For context, the least numerous species (Egyptian goose [*Alopochen aegyptiaca*]) was counted over 85,000 times between 1970 and 2019.

### Species‐specific models

For each species, and in each of the two periods (1970–1994 and 1995–2019), we identified patterns in abundance over time with generalized linear mixed models. We modeled monthly counts at each site as a linear function of year and both climate variables. We also included latitude, longitude, and habitat as explanatory variables and the interaction between each of these and year to describe spatial variation in trends. Month was included as a categorical explanatory variable, interacting (in a pairwise manner) with latitude, longitude, and year, to allow for changes in phenology over space and time. The final explanatory variable was a binary variable to indicate whether the count was recorded as complete or not. We included site as a random factor.

We carried out the modeling in R 4.0.3 (R Core team, 2020). We compared models with a Poisson error distribution to models with a negative binomial error distribution (to account for overdispersion), with and without zero‐inflation terms, implemented with the glmmTMB function of the glmmTMB package (Brooks et al., 2017). Models were consistently better fitting (i.e., lower Akaike information criterion values) with a negative binomial error distribution and a zero‐inflation term, so this model structure was used. We used sum contrasts to compare categorical variables against the mean across all categories. To obtain the coefficients for the last variable in categorical factors, we reran the models with the categorical variables reordered.

### Examining patterns in population trends

We examined whether population trends (i.e., the year coefficient from each species‐specific count models described above) for 1970–1994 and 1995–2019 varied consistently across species in each period with tests of proportion to compare the numbers of species with significant positive and negative trends and between species’ ecological groups, migratory strategies, and breeding locations, also with weighted regression tests (Table 1).

**TABLE 1:** The statistical tests used to examine patterns over time in population trends identified from species‐specific abundance models of 46 wintering waterbird species in the United Kingdom.

Hypothesis	Statistical test
Population trends vary consistently across species in each period.	Test of proportion comparing no. species with significantly positive and negative population trends from 1970 to 1994 (trends_1970–1994_) Test of proportion comparing no. species with significantly positive and negative trends_1995–2019_
Population trends differ between ecological groups, between migratory strategies, and between breeding locations.	A regression of trends_1970–1994_ ≈ −1 + ecological group + migratory strategy + breeding location weighted by 1/(SD_1970–1994_)^2^ to test whether average trends for any species group are significantly different from zero; Gaussian error distribution used and significance of explanatory variables assessed with a likelihood ratio test (LRT) (Chambers & Hastie, 1992) A regression of trends_1995–2019_ ≈ −1 + ecological group + migratory strategy + breeding location, weighted by 1/(SD_1995–2019_)^2^, to test whether average trends for any species group are significantly different from zero. Model structure and LRT as described in the test above.

We then tested whether variations in population trends with climate, habitat, and space were consistent with the patterns expected if they were affected by climate warming, habitat quality changes such as land use and water quality, or changes in phenology. To do this, we repeated the statistical tests set out above (Table 1), replacing the year coefficient response variable (i.e., population trends) with the following coefficients from the species‐specific and period‐specific models: interactions between year and habitat (Bonferroni adjustments used to assess significance in the presence of multiple habitat comparisons) to test for potential associations with habitat; interactions between year and latitude (Bonferroni adjustments used to assess significance of both spatial variables) and between year and longitude to identify large‐scale shifts in abundance consistent with climate‐driven changes in distribution; and climate proxies (winter NAO index and breeding season temperature) to test for annual variation in abundances linked to climate and temperature.

We determined the importance of phenological change driving responses in the models by considering the interactions between month and year. First, we described the phenological pattern in abundance and how this varied over time and spatially by plotting the mean coefficients of the monthly explanatory factors and their interactions with year, latitude, and longitude of migrant and partial migrant species from 1970 to 1994 and from 1995 to 2019. Second, we tested whether population indices could have been reduced by later arrivals or earlier departures by repeating the analyses described in Table 1, but with the interactions between year and the arrival month and departure month as response variables, instead of the year coefficient and removal of nonmigratory species from this analysis. We defined the arrival and departure months for each species as the first and last months in which the mean monthly count across all years was >20% of the mean monthly count in the month with the highest mean count across both periods. The arrival month was September for over 80% of species, and the departure month was March for over 90% of species.

## RESULTS

### Climate warming within and between periods

The mean U.K. temperatures in winter (September–March) and the breeding season (April–August) remained stable between 1970 and 1994 (winter temperature trend = 0.008 [SE 0.010], *t* = 0.54, *p* = 0.595; breeding season temperature trend = 0.018°C [0.014], *t* = 1.26, *p* = 0.22) and between 1995 and 2019 (winter temperature trend = 0.009 [0.019], *t* = 0.46, *p* = 0.647; breeding season temperature trend = 0.014°C [0.013], *t* = 1.12, *p* = 0.274). However, there were higher mean temperatures from 1995 to 2019 than from 1970 to 1994 in winter (mean temperatures = 6.8°C and 6.1°C, respectively, *t* = 4.06, *p* < 0.001) and during the breeding season (mean temperatures = 12.5°C and 11.7°C, respectively, *t* = 5.80, *p* < 0.001) (Appendix S2).

### Examining patterns in population trends: patterns across all species

We modeled counts for 46 wintering waterbird species from 1970 to 1994 and from 1995 to 2019. Data were not available for moorhen (*Gallinula chloropus*) from 1970 to 1994.

**FIGURE 1:**
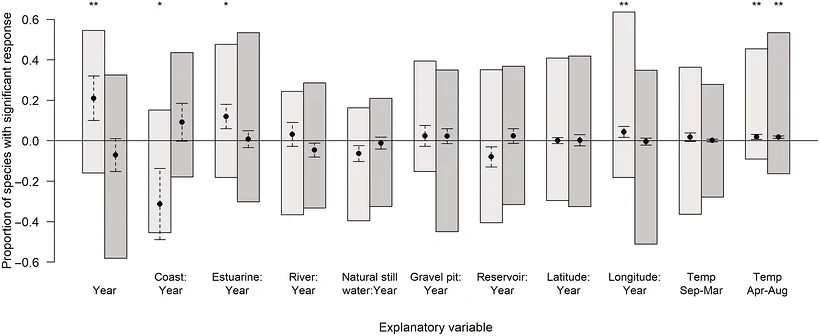
The proportion of species of U.K. wintering waterbirds with significant positive and negative relationships between counts and explanatory variables (circles, mean of coefficients weighted by 1/variance; dashed lines, 95% confidence intervals) from 1970 to 1994 (pale bars) and 1995 to 2019 (dark bars) (asterisks, proportion of species with significantly positive and negative responses significantly different from 50% [***p < 0.001; **p < 0.01; *p < 0.05).

Most species (54.5%) showed significant, positive population trends from 1970 to 1994, compared with only 15.9% showing significant, negative trends during this period (Table 2; Figure 1). These trends did not vary significantly between habitats after Bonferroni adjustments to account for comparing multiple habitats. There was a positive effect of longitude on trend, indicative of more positive population trends in the east of the United Kingdom than the west (Table 2; Figure 1). This contrasts with 1995–2019, when mean population trends were stable on average, with 58% of species showing significantly declining population trends and 33% of species showing significantly increasing trends (Table 2; Figure 1). In this later period, there were no significant interactions between habitat or location and year (Table 2; Figure 1).

**TABLE 2:** Percentage of U.K. wintering waterbird species with negative and positive coefficients in species‐specific abundance models and the difference in means between species groups in two periods.

		Difference in means between species groups (*F*, *p*)^a^		
Coefficient	Species with significant negative or positive coefficients (%) (χ^2^, *p*)	Ecological groups (five groups)	Migration strategy (three groups)	Breeding location (four groups)
Year	15.9, 54.5 (8.3, 0.004)^b^	0.54, 0.711	2.21, 0.123	0.21, 0.811
	58.1, 32.6 (2.6, 0.109)	5.2, 0.002	0.71, 0.496	0.6, 0.556
Open coast: Year	45.5, 15.2 (4, 0.044)	0.62, 0.654	1.98, 0.171	5.2, 0.005
	17.9, 43.6 (3.4, 0.066)	2.89, 0.037	0, 0.993	1.16, 0.34
Estuarine: Year	18.2, 47.7 (5, 0.026)	4.91, 0.003	1.97, 0.154	1.46, 0.247
	30.2, 53.5 (2.2, 0.134)	4.6, 0.004	0, 0.962	0.22, 0.881
River or marsh: Year	36.6, 24.4 (0.6, 0.424)	4.07, 0.008	3.54, 0.069	1.51, 0.229
	33.3, 28.6 (0, 0.845)	2.03, 0.113	1.24, 0.274	2.18, 0.106
Natural inland still water: Year	39.5, 16.3 (3.4, 0.066)	4.82, 0.003	1.21, 0.31	0.04, 0.956
	32.6, 20.9 (0.7, 0.404)	0.57, 0.684	12.86, 0.001	5.37, 0.009
Gravel pit: Year	15.2, 39.4 (2.7, 0.099)	3.07, 0.032	0.19, 0.827	0.17, 0.846
	45, 35 (0.3, 0.596)	6.07, 0.001	0.37, 0.691	0.81, 0.452
Reservoir: Year	40.5, 35.1 (0, 0.85)	4.74, 0.004	2.26, 0.122	0.8, 0.459
	31.6, 36.8 (0, 0.845)	2.04, 0.111	1.26, 0.297	0.29, 0.748
Latitude: Year	29.5, 40.9 (0.5, 0.472)	7.61, <0.001	0.02, 0.878	0.7, 0.559
	32.6, 41.9 (0.3, 0.596)	0.25, 0.907	0.55, 0.582	0.3, 0.745
Longitude: Year	18.2, 63.6 (10, 0.002)	9.05, <0.001	0.14, 0.715	0.68, 0.569
	51.2, 34.9 (1, 0.324)	1.42, 0.246	0.32, 0.727	0.04, 0.962
Temperature (September–March)	36.4, 36.4 (0, 1)	2.85, 0.037	0.6, 0.553	0.17, 0.845
	27.9, 27.9 (0, 1)	2.78, 0.041	1.66, 0.203	0.3, 0.746
Temperature (April–August)	9.1, 45.5 (9.4, 0.002)	0.16, 0.959	0.24, 0.628	0.61, 0.612
	16.3, 53.5 (7.5, 0.006)	3.78, 0.012	8.62, 0.006	6.96, 0.003
Autumn: Year (excluding residents)	44.8, 34.5 (0.2, 0.677)	1.25, 0.305	2.43, 0.132	9.12, 0.001
	28.6, 35.7 (0.1, 0.814)	0.82, 0.452	0.57, 0.457	0.18, 0.835
Spring: Year (excluding residents)	27.6, 34.5 (0.1, 0.814)	1.06, 0.362	18.75, <0.001	17.51, <0.001
	21.4, 39.3 (0.9, 0.332)	0.95, 0.431	1.7, 0.204	0.06, 0.805

*Note*: Data from 1970 to 1994 in upper rows associated with each coefficient are for 45 wintering waterbird species, and data from 1995 to 2019 in lower rows are for 46 species.

^a^ The *F* and *p* values are from likelihood ratio tests of linear regression models.

^b^ Significant results: *p* < 0.05 or *p* < 0.0083 for habitat variables or *p* < 0.025 for spatial variables due to Bonferroni adjustments.

For many species, there were significant positive associations between abundance and U.K. breeding season temperature in both periods (45.5% and 53.5% with significantly positive associations between abundance and temperature in the early and late periods, respectively, compared with 9.1% and 16.4% with significantly negative associations) (Table 2; Figure 1). Similar numbers of species had populations that correlated positively and negatively with winter temperatures. There was no evidence of consistent changes across all migrant species (including partial migrants) in arrival month (autumn year interaction) or departure month (spring year interaction) (Table 2).

### Patterns in population trends

From 1970 to 1994, population trends did not vary significantly between species based on ecological group, migration strategies, or breeding location (Table 2). From 1995 to 2019, population trends varied between species of different ecological groups. In particular, population trends were more positive than average for geese and swans and more negative than average for waders (Table 3; Figure 2a).

**TABLE 3:** Mean estimates of correlation between species abundance and model coefficients within ecological groups for factors with significant differences between ecological groups (Table 2) in two periods.

	Ecological group				
Coefficient	Goose and swan mean [SE] (*t*, *p*)	Dabbling duck and rail mean [SE] (*t*, *p*)	Diving duck mean [SE] (*t*, *p*)	Piscivore mean [SE] (*t*, *p*)	Wader mean [SE] (*t*, *p*)
	
Year	0.302 [0.123] (2.5, 0.018)^a^	−0.066 [0.052] (−1.3, 0.213)	−0.198 [0.099] (−2, 0.053)	0.134 [0.102] (1.3, 0.195)	−0.246 0.072] (−3.4, 0.002)
Open coast: Year					
					
Estuarine: Year	0.144 [0.071] (2, 0.048)	0.127 [0.044] (2.8, 0.007)	0.2 [0.064] (3.1, 0.003)	0.142 [0.049] (2.9, 0.006)	−0.353 [0.112] (−3.1, 0.003)
	−0.057 [0.064] (−0.9, 0.379)	0.024 [0.029] (0.8, 0.403)	−0.093 [0.053] (−1.7, 0.09)	−0.11 [0.05] (−2.2, 0.036)	0.111 [0.036] (3.1, 0.004)
River or marsh: Year	0.006 [0.07] (0.1, 0.936)	0.015 [0.041] (0.4, 0.713)	0.18 [0.062] (2.9, 0.007)	0.048 [0.057] (0.8, 0.408)	−0.334 [0.114] (−2.9, 0.006)
	
Natural inland still water: Year	−0.09 [0.053] (−1.7, 0.097)	−0.111 [0.031] (−3.6, 0.001)	0.005 [0.044] (0.1, 0.909)	−0.06 [0.027] (−2.3, 0.03)	0.6 [0.176] (3.4, 0.002)
	
Gravel pit: Year					
	−0.024 [0.046] (−0.5, 0.603)	0.062 [0.021] (2.9, 0.006)	0.113 [0.039] (2.9, 0.006)	−0.101 [0.042] (−2.4, 0.02)	−0.083 [0.045] (−1.8, 0.073)
Reservoir: Year	0.034 [0.068] (0.5, 0.62)	−0.141 [0.039] (−3.6, 0.001)	−0.058 [0.055] (−1.1, 0.294)[	−0.087 [0.033] (−2.6, 0.013)	0.791 [0.237] (3.3, 0.002)
	
Latitude: Year	0.011 [0.017] (0.7, 0.502)	−0.017 [0.01] (−1.7, 0.106)	−0.025 [0.012] (−2.1, 0.043)	−0.02 [0.02] (−1, 0.316)	0.06 [0.013] (4.7, <0.001)
	
Longitude: Year	−0.001 [0.03] (0, 0.967)	0.021 [0.018] (1.2, 0.247)	−0.016 [0.023] (−0.7, 0.501)	0.017 [0.033] (0.5, 0.6)	0.144 [0.02] (7.2, <0.001)
	
Temperature (September–March)	0.027 [0.027] (1, 0.313)	0.007 [0.017] (0.4, 0.693)	−0.015 [0.021] (−0.7, 0.475)	0.104 [0.03] (3.5, 0.001)	0.019 [0.022] (0.9, 0.391)
	0 [0.009] (−0.1, 0.958)	0.003 [0.005] (0.5, 0.616)	−0.023 [0.009] (−2.6, 0.013)	0.013 [0.009] (1.5, 0.137)	0.014 [0.01] (1.4, 0.174)
Temperature (April–August)					
	0.01 [0.009] (1.2, 0.252)	0.024 [0.005] (4.7, <0.001)	0.008 [0.009] (0.9, 0.368)	0.022 [0.008] (2.6, 0.013)	0.012 [0.01] (1.2, 0.236)
Autumn: Year (excluding residents)					
					
Spring: Year (excluding residents)					
					

*Note*: Data from 1970 to 1994 in upper rows associated with each coefficient are for 45 wintering waterbird species, and data from 1995 to 2019 in lower rows are for 46 species. Blank lines indicate habitats with no significant differences between ecological groups.

^a^ Significant results (*p* < 0.05).

FIGURE 2The mean of coefficients from species‐specific models of waterbirds (weighted by 1/variance ± 95% confidence intervals from models of coefficients varying among groups assessed by LRT) within species groups by ecological group, migratory strategy, and breeding location, for which significant differences were found between groups: (a) population trends (i.e., year coefficient); (b–g) population trends in specific habitats relative to other habitats (mean habitat trend = mean population trend + relative population trend on habitat); (h) latitudinal or (i) longitudinal gradient to population trends (i.e., interaction between latitude or longitude and year); and (j, k) association between abundance and mean temperature in the United Kingdom from September to March and from April to August prior to the winter counts (circles and solid lines, coefficients for models with data from 1970 to 1994; triangles and dashed line, coefficients for models with data from 1995 to 2019; percentages in [b–g], mean percentage of counts in the habitat across all species in the ecological group and in both periods).

**Figure:**
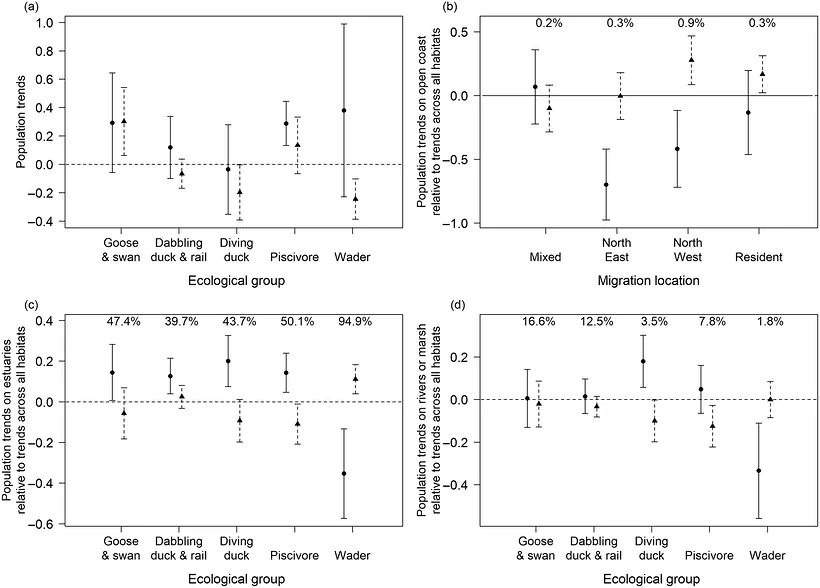

**Figure:**
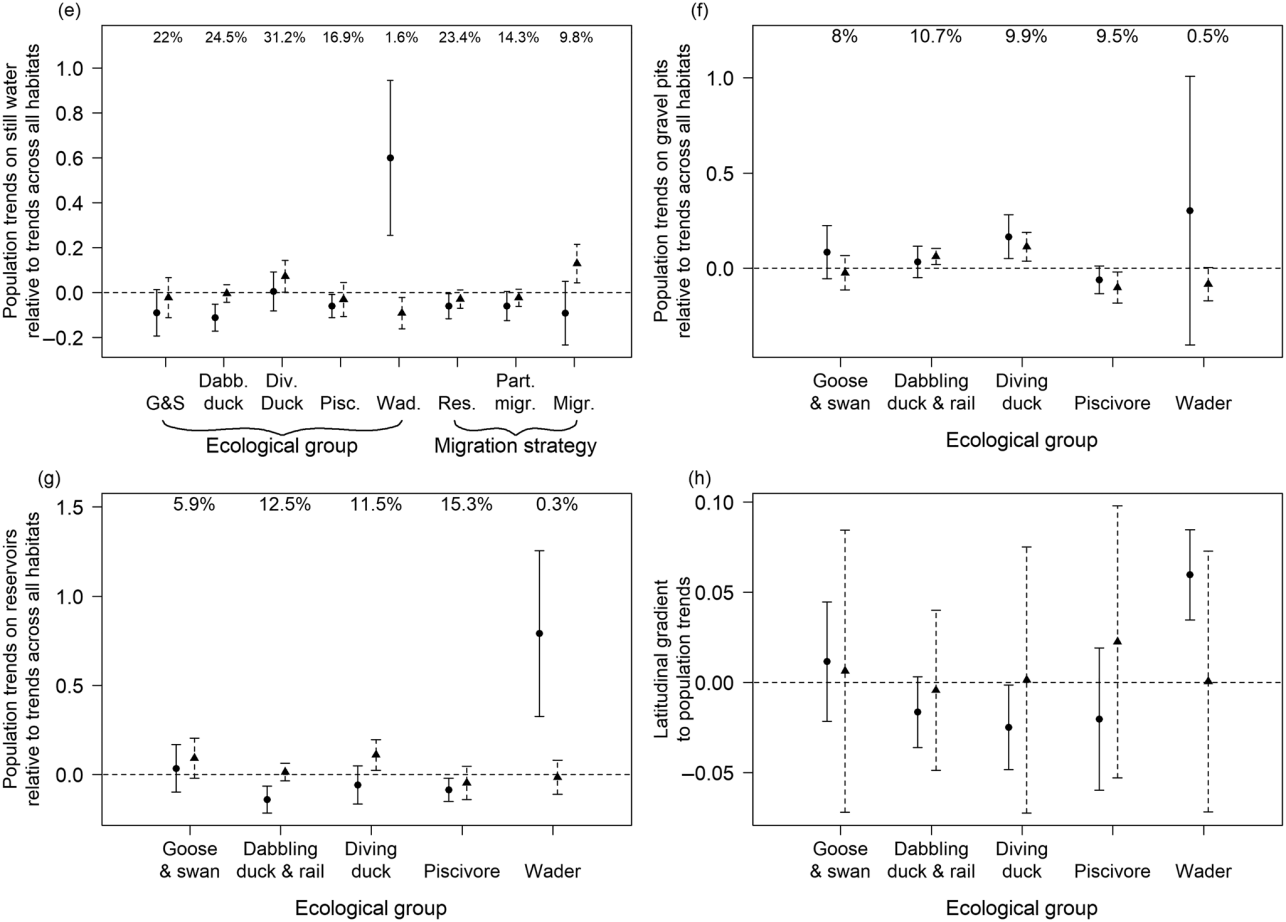

**Figure:**
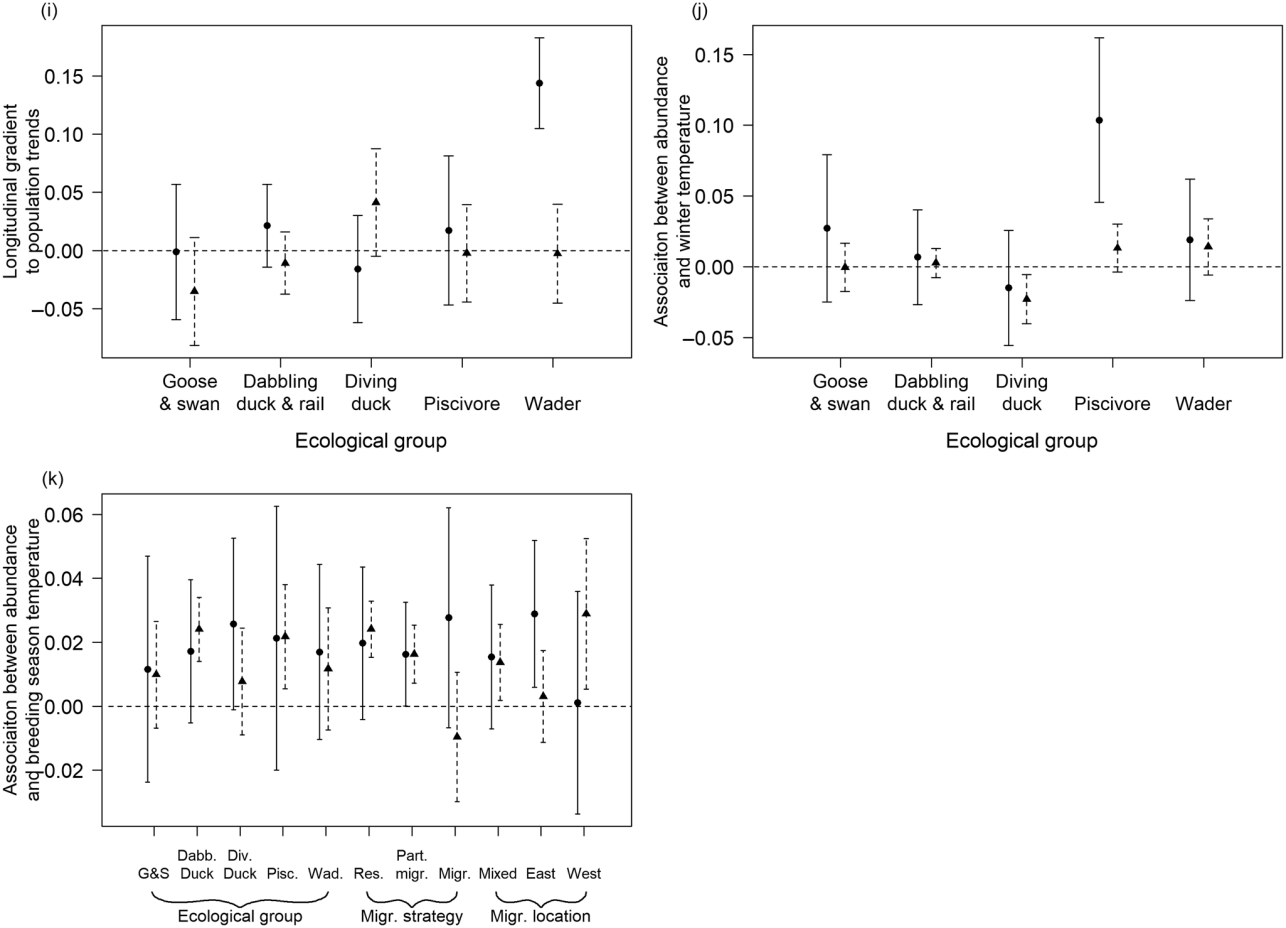

### Variation between habitats

There was some variation in habitat‐specific population trends in all habitats in one or both periods, generally between ecological groups (Table 2). From 1970 to 1994, wader population trends were stable overall (Figure 2a), but were more negative on estuaries (Figure 2c) and rivers or marshes (Figure 2d; Table 3), although this variation between habitats did not result in significant declines on these habitats (habitat trend = mean weighted year trend + relative habitat trend = 0.023 [SE 0.303], *t* = 0.08, *p* = 0.939 and −0.0189 [0.257], *t* = −0.07, *p* = 0.942 for estuaries and rivers or marshes, respectively). Wader trends during this time had no overall significant increases but were more positive on natural inland still water (Table 3; Figure 2e) and reservoirs (Figure 2g), leading to significant increases on natural inland still water (habitat trend = 0.950 [0.412], *t* = 2.3, *p* = 0.027) and insignificant increases on reservoirs (habitat trend = 1.180 [0.588], *t* = 2.01, *p* = 0.053).

Goose and swan population trends from 1970 to 1994 were more positive on estuaries than on other habitats (Table 3; Figure 2c), leading to positive trends (estuarine habitat trend = 0.442 [SE 0.183], *t* = 2.42, *p* = 0.020). Dabbling duck population trends from 1970 to 1994 were more positive on estuaries than on other habitats (Figure 2c; Table 3), leading to population increases (estuarine habitat trend = 0.240 [0.115], *t* = 2.09, *p* = 0.043), and their population trends were more negative on natural inland still water (Figure 2e) and reservoirs (Figure 2g; Table 3), although these did not result in significant declines (habitat trend = 0.007 [0.094], *t* = 0.07, *p* = 0.944 and −0.029 [0.128], *t* = −0.22, *p* = 0.824 on natural inland still water and reservoirs, respectively).

Diving duck population trends from 1970 to 1994 were more positive on estuaries (Figure 2c) and rivers or marshes (Figure 2d; Table 3), although these did not lead to significant increases (habitat trend = 0.173 [SE 0.165], *t* = 1.05, *p* = 0.301; 0.118 [0.137], *t* = 0.86, *p* = 0.394). The increasing piscivore population trends during this time were more positive on estuaries (Figure 2c) (habitat trend = 0.443 [0.106], *t* = 4.26, *p* < 0.001) and more negative on natural inland still water (Figure 2e) and reservoirs (Figure 2g) than other habitats (Table 3), although piscivore population trends were still positive on these habitats (habitat trend = 0.236 [0.074], *t* = 3.17, *p* = 0.003 and 0.208 [0.101], *t* = 2.06, *p* = 0.047 for natural inland still water and reservoirs, respectively). In this period (1970–1994), population trends of species that migrated to Scandinavia, northwestern Europe, and Siberia (relative population trend = −0.698 [0.142], *t* = −4.9, *p* < 0.001) or to Iceland, Greenland, and North America (relative population trend = −0.418 [0.154], *t* = −2.7, *p* = 0.011) had more negative population trends if they were wintering in open coastal habitats (Figure 2b).

From 1995 to 2019, the overall declines in wader populations were significantly less negative on estuaries than on other habitats (Table 3; Figure 2c), although wader populations still declined on estuaries (habitat trend = −0.140 [SE 0.060], *t* = −2.34, *p* = 0.025). Piscivore population trends were more negative on estuaries than on other habitats (Table 3; Figure 2c), although still not significantly changing (habitat trend = 0.019 [0.083], *t* = 0.22, *p* = 0.824). On gravel pits, dabbling duck and diving duck population trends were significantly more positive than other habitats, although still stable (habitat trends = −0.009 [0.061], *t* = −0.15, *p* = 0.881 and 0.074 [0.113], *t* = 0.65, *p* = 0.517 for dabbling ducks and diving ducks, respectively), whereas piscivore gravel pit population trends were more negative than other habitats (Table 3; Figure 2f), although still stable (habitat trends = 0.074 [0.121], *t* = 0.613, *p* = 0.544). In this period (1995–2019), migrant population trends were more positive on natural inland still water (difference in population trend for migrants = 0.129 [0.044], *t* = 2.9, *p* = 0.006) (Figure 2e).

### Spatial variation

Spatial variation in population trends differed between ecological groups from 1970 to 1994 but not from 1994 to 2019 (Table 2). From 1970 to 1994, waders had more positive wintering population trends in the northern United Kingdom than in the southern United Kingdom (Table 3; Figure 2h,i). Diving ducks had more negative wintering population trends in the northern United Kingdom than in the southern United Kingdom during this period (Table 3; Figure 2h).

### Variation linked to weather

Species’ responses to U.K. winter temperature varied between ecological groups in both periods (Table 2). From 1970 to 1994, piscivores had a more positive response to warm winters than other groups, and from 1995 to 2019, diving ducks had a negative response to warm winters (Table 3; Figure 2j). Species responses to breeding season temperature varied with ecological group, migration strategy, and migration location from 1995 to 2019; dabbling duck and piscivore populations trends were positively associated with temperature (Table 3; Figure 2k), as were trends for species that were resident in the United Kingdom throughout the year (0.024 [SE 0.005], *t* = 5.4, *p* < 0.001), partial migrants (0.016 [0.005], *t* = 3.5, *p* = 0.001), migrants with mixed locations (0.014 [0.006], *t* = 2.3, *p* = 0.03), and migrants that migrated to Iceland, Greenland, and North America (0.029 [0.012], *t* = 2.4, *p* = 0.021) (Figure 2k).

### Phenological variation

**FIGURE 3:**
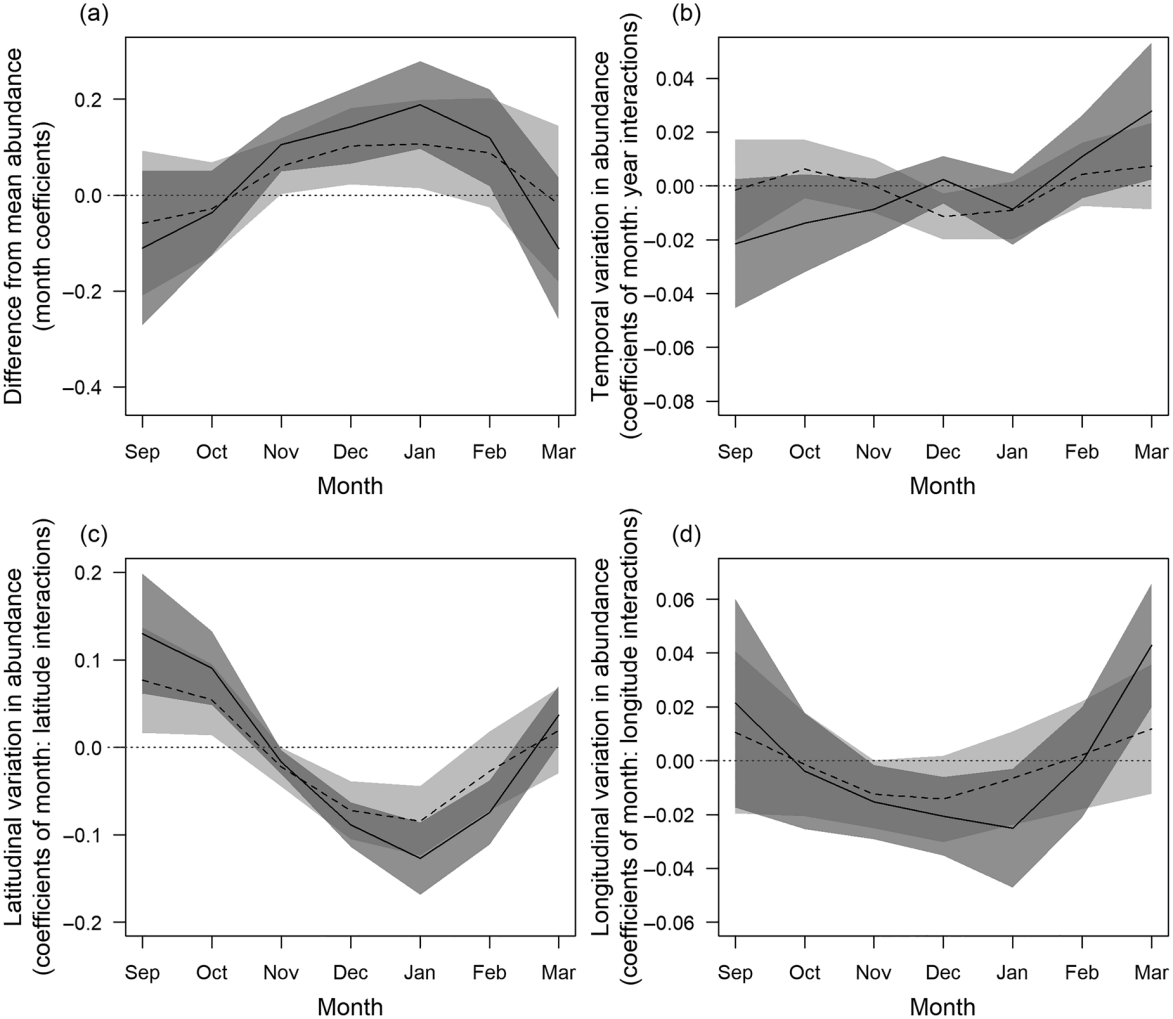
The pattern of (a) phenology (monthly coefficients in species‐specific models), (b) phenological change (month:year interactions in species‐specific models), and (c, d) spatial pattern in phenology (month:latitude and month:longitiude interactions in species‐specific models) across waterbirds wintering in the United Kingdom (resident species excluded) based on means from 1970 to 1994 (solid lines) and 1995 to 2019 (dashed lines) (shading, 95% confidence intervals).

In general, species had lowest abundances in the early wintering period (September), peaking in January and declining a little by March (Figure 3). There were no trends in arrival or departure months apparent across all full and partial migrants (Table 2).

**FIGURE 4:**
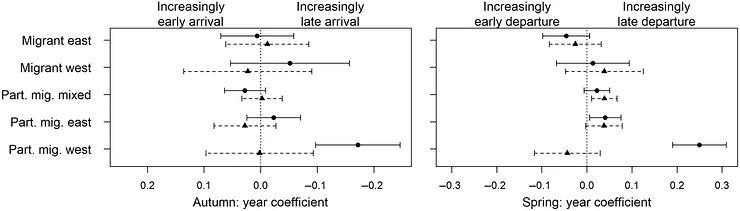
Mean (95% confidence intervals) change in arrival (autumn) and departure (spring) dates of waterbirds from species‐specific models for species divided by migratory strategy and migration location from 1970 to 1994 (circles) or 1995 to 2019 (triangles).

In both periods, changes in apparent arrival and departure months varied depending on species’ migration strategy and location (Figure 4). In particular from 1970 to 1994, species with partial migration breeding in Iceland, Greenland, and North America (i.e., oystercatcher [*Haematopus ostralegus*], golden plover [*Pluvialis apricaria*], and redshank [*Tringa tetanus*]) were increasingly less abundant in the arrival month (autumn: year coefficient = −0.172 [SE 0.038], *t* = −4.5, *p* = 0.001) and increasingly more abundant in the departure month (spring: year coefficient = 0.240 [0.0303], *t* = 8.2, *p* < 0.001) (Figure 4). This indicates that the duration of occurrence of these species in the United Kingdom, on average, remained largely the same, but may have shifted later (Figure 4). No other groups of migrants appeared to have significantly changed their arrival times in either period. Two other groups appeared to delay their departure times a little (or smaller proportions of the species were migrating); partial migrants breeding in Scandinavia, northwestern Europe, and Siberia (i.e., pochard [*Aythya ferina*], tufted duck [*Aythya fuligula*], avocet [*Recurvirostra avosetta*], lapwing [*Vanellus vanellus*], and ringed plover [*Charadrius hiaticula*]) became increasingly abundant in the spring departure month from 1970 to 1994 (0.041 [0.018], *t* = 2.3, *p* = 0.031), whereas from 1995 to 2019, partial migrants with mixed breeding grounds (shoveler [*Spatula clypeata*], gadwall [*Mareca strepera*], wigeon [*Mareca Penelope*], mallard [*Anas platyrhynchos*], pintail [*Anas acuta*], and teal [*Anas crecca*]) became more abundant in the spring departure month (0.039 [0.0143], *t* = 2.7, *p* = 0.013) (Figure 4).

## DISCUSSION

There has been a very clear change in the population trends of U.K. wintering waterbirds over the past 50 years. In the early half of the 50‐year period (1970–1994), most wintering waterbird population trends were positive, whereas during the latter half of the 50 years (1995–2019), population trends became more negative, particularly for waders. Trends remained positive for geese and swans. The extent to which our results are consistent with different potential drivers of change is elaborated below based on interactions between population trends of different groups of species and habitat and climate. However, changes in climate could also influence habitat use (Burton et al., 2023). The drivers of population change are likely to vary between species, and for most species, multiple drivers may be affecting populations.

### Association between waterbird trends and habitat

From 1970 to 1994, wintering waterbird population increases were most apparent in estuaries for all ecological groups except waders. For most species in this study, estuaries support most of their population, making the trends on estuaries particularly important. The population trends in the later period, from 1995 to 2019, were generally more negative than in the early period. During this period, wader populations declined on estuaries, although their declines were less substantial (but still significant).

There are a range of possible explanations for the increases in wintering waterbird populations from 1970 to 1994, being most apparent in estuaries and less apparent in coastal habitats. One potential driver for the pattern of trends is changes in water quality, although it is likely that other factors also play a role.

In the United Kingdom, water quality generally worsened (i.e., declining oxygen and increasing nitrogen and phosphorus) until legislative changes in the early 1990s, after which it improved (Maier et al., 2009). Many estuarine wintering waterbirds benefit from nitrogen‐ and phosphorus‐rich agricultural runoff and sewage associated with low water quality (Burton et al., 2002; Pringle & Burton, 2017), so water quality improvements could have played a role in the slowing of estuarine population increases. In freshwater, the relationship between water quality and wintering waterbird populations is inconsistent (Davies & Sylvester‐Bradley, 1995; Møller & Laursen, 2015) because, in some cases, macroinvertebrate prey declines were linked to water quality declines, as seen from 1970 to 1994 (Durance & Ormerod, 2009; Vaughan & Ormerod, 2012). In other instances, macroinvertebrate prey declines have been linked to water quality improvements (Marsden & Bellamy, 2000; Schummer et al., 2008; Tománková, 2013; Tománková et al., 2014). Wader populations are negatively associated with water quality due to declines in invertebrate prey (Pringle & Burton, 2017), so declines from 1995 to 2019 could be, in part, linked to water quality improvements.

Another potential driver of increasing counts from 1970 to 1994 could be that optical equipment became significantly more powerful and affordable, which could have increased counts, particularly in estuaries where counts were often higher than in other habitats. However, the strong positive correlations between counts and longitude and temperature suggest that the positive trends from 1970 to 1994 are likely to be genuine.

### Associations with climate warming

Most species’ abundances showed positive correlations with U.K. breeding season temperature in both 25‐year periods. Resident species particularly benefitted from increasing breeding season temperatures, consistent with studies of land birds where resident species have tended to benefit more from recent warming than long‐distance migrants (Howard et al., 2020; Pearce‐Higgins et al., 2015). The impacts of climate warming on resident species could be through direct effects on breeding conditions (Pearce‐Higgins et al., 2015) but may also be linked to indirect ecological mechanisms (Ockendon et al., 2014), such as changes in food resources, disease, predator populations, or competitors. The positive correlations between migratory species and U.K. breeding season temperatures could be due to the impact of breeding season temperature on food resources the following winter, or due to U.K. breeding season temperatures correlating with Arctic breeding season weather. Increasing temperatures may therefore partly account for the increase in population trends in most wintering waterbird species from 1970 to 1994.

Across all species, there were still generally positive associations between abundance and U.K. breeding season temperature from 1995 to 2019, but the benefits of warm years were lower in the later period and disappeared for migrant species, particularly those migrating to Scandinavia, northwestern Europe, or Siberia. Populations of migratory species breeding at higher latitudes are likely to be particularly affected by climate warming on those northern breeding grounds (Boyd, 2013; Clausen & Clausen, 2013; Layton‐Matthews et al., 2020; Lindström & Agrell, 1999; Rehfisch & Crick, 2003). Although temperature increases on the Arctic breeding grounds may bring benefits, such as increasing insect and other food availability, there could also be negative impacts, such as breeding habitats disappearing as vegetation zones move northward or predator abundance increases (Layton‐Matthews et al., 2020; Lindström & Agrell, 1999). Some changes may be an immediate response to increased temperatures (e.g., insect abundance), whereas others could take longer to occur (e.g., shifting vegetation zones). This shifting balance of positive and negative impacts of warming could explain why the impact of warming became less positive over time. More research into the mechanisms behind the correlation between temperature and waterbird abundance would help to predict future population changes.

Winter temperatures did not consistently affect waterbird population trends, but piscivores appeared to have benefited from increasing winter temperatures from 1970 to 1994 because of very large population increases in the colonizing little egret (*Egretta garzetta*). Diving duck populations were negatively associated with increasing winter temperatures from 1995 to 2019. Previously, population increases until the mid‐1990s have been linked to increases in the winter NAO index (Hurrell et al., 2020), which generally brings warmer, wetter winters to the United Kingdom and increases waterbird survival, particularly in the colder east (Austin & Rehfisch, 2005; Austin et al., 2000; Cook et al., 2021; Maclean et al., 2008; Pavón‐Jordán et al., 2019). Although we did not find strong evidence linking abundance and winter temperatures, population trends were more positive in the colder east than the west from 1970 to 1994, giving some support for this link.

The population shift from the warmer west to the colder east from 1970 to 1994 was most apparent in waders. Higher winter temperatures may be particularly beneficial for waders because they feed on invertebrates, which are more abundant on the sandier east of Britain, but they may experience high mortality in severe winter conditions (Austin & Rehfisch, 2005; Austin et al., 2000). However, no eastward or northward shift was detected in waders during 1995–2019, when waders were declining. Declines of waders’ shellfish prey from the 1990s due to overfishing, particularly in eastern England, may have reduced any climate‐driven eastward population shift (Atkinson et al., 2003, 2010; Bowgen et al., 2022). The benefits of warming winter temperatures for waders may be offset by declines driven by changing breeding season weather. Research into wader declines shows that wader productivity and population sizes are loosely linked to predation risk and availability of breeding habitat, both of which may become less optimal for waders due to climate change (Amar et al., 2011; Cook et al., 2021; Franks et al., 2018; Kubelka et al., 2018; Roodbergen et al., 2012; Wauchope et al., 2017; Woodward et al., 2021).

For Arctic and continental breeding species, warming temperatures may have reduced the distances that individuals need to migrate to avoid cold‐induced high mortality (Cook et al., 2021) (i.e., short stopping). Short stopping could lead to U.K. winter population increases or declines, depending on whether a species is increasingly overwintering in the United Kingdom from warmer parts of Europe, such as Spain, or increasingly overwintering in colder parts of Europe from the United Kingdom. There is little evidence of waterbirds short stopping in the United Kingdom from warmer European wintering grounds (Nightingale, 2023; Woodward et al., 2024), whereas there is considerable evidence of U.K. wintering waterbird abundance declining throughout the United Kingdom and in the southwest due to short stopping (Burton et al., 2020, 2023; Pavón‐Jordán et al., 2019; Podhrázský et al., 2017). However, short stopping may be a result of range contractions rather than solely range shifts due to climatic debt (Gaget et al., 2020; Marchowski et al., 2020). Waders, the group experiencing the greatest recent U.K. declines in our analysis, are generally declining elsewhere in Europe (Roodbergen et al., 2012; Silva‐Monteiro et al., 2021). An indicator of Arctic breeding waterbird populations, examining many of the same species as in this study, showed a similar pattern of increasing populations until the mid‐1990s, followed by populations stabilizing or declining over the past 25 years (Thaxter et al., 2025), suggesting that U.K. declines are likely to reflect broader flyway population trends. More information on flyway population trends and spatial distribution, particularly in Eastern Europe and Russia, would help determine the extent to which wintering ranges of migratory species were contracting and shifting.

We considered that apparent declines in wintering waterbird populations since the mid‐1990s could result from changes in phenology. Later arrival and earlier departure from the United Kingdom could reduce wintering trends if counts drop in the autumn and spring (Frost, 2010). Partial migrants that migrated to Iceland, Greenland, and North America did show increasingly late arrival in the United Kingdom from 1970 to 1994, but they also showed increasingly late departures, which would not influence wintering population estimates. This pattern of delayed autumn and spring migration is consistent with other research on migratory waterbirds (Lehikoinen & Jaatinen, 2012), although earlier spring migration leading to short staying on wintering grounds is more commonly reported for Arctic waterbirds (Guillemain et al., 2013; Lameris et al., 2018; Lehikoinen et al., 2019; Rainio et al., 2006). From 1995 to 2019, many partial migrants departed increasingly late (or fewer migrated), which could lead to positive wintering trends but cannot explain the generally more negative trends during this period.

To conclude, although short stopping may have driven some of the decline in U.K. wintering waterbird populations, climate warming was still linked to positive trends from 1995 to 2019 for most species, particularly residents and partial migrants. It is therefore unlikely that climate warming in the United Kingdom directly caused the shift away from population rises after the mid‐1990s.

### Population indices influenced by movements to less‐monitored areas

Apparent trends in wintering waterbird indices may be caused by variation in population trends between well‐monitored habitats and regions of the United Kingdom compared with less‐monitored habitats and areas, biasing population estimates. There were more positive population trends in the best‐monitored habitat type, estuaries, than in the less‐monitored habitats from 1970 to 1994, which could have inflated some U.K. wintering waterbird population trend estimates. Likewise, population trends were higher in the better‐monitored east than in the west from 1970 to 1994. However, the populations increasing during these 25 years were still apparent in our analyses, which accounted for potential differences in trends between habitats and regions. There were no consistent spatial patterns in trends or differences between habitats from 1995 to 2019, but species‐specific patterns could bias population trends and should be considered in the future when population trends are calculated.

### Other factors

There are many other potential drivers of the early increase in wintering waterbirds including the benefits of increased restrictions on wildfowl hunting (Fox & Madsen, 2017; Taylor & Dodd, 2013), which contributed to increases in abundance in species such as curlew (*Numenius arquata*) (Woodward et al., 2021). The recent rise in geese and swan populations could be a legacy of recovering from overhunting and may also be linked to refuge provision and the increasing extent of improved grassland and other agricultural land, which provided high‐quality forage for these species (Fox & Madsen, 2017; Mason et al., 2018). Other changes may be linked to increasing waterbird populations before the mid‐1990s. Toxins such as heavy metals and pesticides are likely to have declined in estuaries from the 1970s, which could have benefitted wintering waterbirds (Power et al., 1999; Vane et al., 2020). Lead contamination was reduced by the regulation of lead fishing weights, introduced in the late 1980s and thought to be responsible for increases in wintering waterbird populations, such as mute swan (*Cygnus olor*) populations (Wood et al., 2019).

We confirmed that most U.K. wintering waterbird populations increased from 1970 to 1994. Population trends became more negative from 1995 to 2019. Declines were seen in waders in particular, whereas increases continued in geese and swans (Frost et al., 2021), potentially linked to recovery from limits associated with hunting and lead pollution and to the exploitation of productive farmed habitats. We found some indications that the initial increase may be partly associated with increasing temperatures. Significant variation between habitats is worthy of further investigation. They may indicate possible impacts of changes in water quality (although we did not measure this directly), although other potential drivers include increases in hunting legislation and reductions in lead contamination and other heavy metals.

More recent declines in wintering waterbirds were apparent from 1994 to 2019, particularly in waders. This was during a time of improvements in water quality, following legislation introduced in the early 1990s, which may have reduced macroinvertebrate prey abundance in some waterbodies, particularly estuarine habitats (Burton et al., 2002; Tománková, 2013; Pringle & Burton, 2017). The wintering waterbird declines did not appear linked to U.K. winter or breeding season temperature change. However, we found some evidence of species shifting their wintering range northward and eastward to colder wintering ranges outside the United Kingdom as temperatures rose, which could contribute to these declines. Waders are experiencing global declines (Roodbergen et al., 2012; Silva‐Monteiro et al., 2021), so it is likely that there are also other factors driving declines in the United Kingdom.

Although we identified some possible drivers of the population declines in some U.K. wintering waterbirds since the mid‐1990s, there is an urgent need to understand the causes of these declines further, particularly in light of the international importance of the U.K.’s wintering waterbird populations.

## Supporting information



Supplementary Materials.

Supplementary Materials.
