# Supplementary material for: Habitat and spatial trends of U.K. wintering waterbird populations over 50 years
Source: Conserv Biol. 2026 May 14;40(5):e70310. doi: 10.1111/cobi.70310 (PMC13613564; doi:10.1111/cobi.70310)
Supplement: Supplementary file 1 [file COBI-40-e70310-s001.docx]

**Supplementary Material 2: Weather variables**

*Exploratory modelling to identify weather variables*

We identified the NAO index and UK mean temperature over the winter months of the WeBS survey (September – March) as climate proxies that could allow us to examine the link between climate and wintering waterbird abundance in the UK. The NAO index represents the large-scale climatic conditions for the North Atlantic Ocean (Hurrell, 1995) and were downloaded from Hurrell et al (2020). The UK September-March temperature was taken from the mean monthly UK temperatures derived from 1km gridded temperatures (Met Office, 2018; Hollis et al., 2019).

We also identified breeding season weather (April – August) as a factor that could affect waterbird abundance. As some UK wintering birds are resident species and other migrate around northern Europe and the Arctic, we considered the UK temperature and the NAO index over the breeding season as potentially important climate proxies. As these variables correlate with each other we first carried out explanatory modelling to identify which of these variables was most linked to population abundance.

We then examined whether there were trends in the selected weather variables from 1970 – 1994 and from 1995 – 2019 and whether there were differences in weather between these two 25-year periods.

*Methods*

To identify the most appropriate breeding season and winter weather variables to include in our study, we modelled counts (for each of the 46 species included in this study separately) from 1970 – 2019 against one of each of the six weather variables: NAO index and mean UK temperature from the September-March of monitoring (long winter), from November-February (short winter) and from the April-August prior to monitoring (breeding season). We compared AIC values of the resulting models for each species to identify the winter variable and breeding season variable that varied most closely with population abundance for the majority of species. For breeding season variables, we examined which variable explained population abundance variation most closely for resident, partial migrant and migrant species.

The models were structured as stated in the paper methods (general linear mixed models with negative binomial distributions, and zero-inflation factors) and all of the other explanatory variables were also included (month, year, latitude, longitude, habitat, completeness) without any interactions. We also included breeding season temperature for models used to compare the fit of winter weather variables and UK winter temperature in the models used to compare the fit of breeding season weather variables.

To compliment the interpretation of our results we used linear models to test whether there were trends in the selected weather variables within each of the time periods (1970 – 1994 and 1994 – 2019), and we used t-tests to compare the means between the time periods.

*Results*

The winter weather variable that correlated more closely with abundance for most species (80.4%) was the UK winter temperature from September - March (Table SM1).

The UK mean breeding season temperature varied more closely to population abundance than the breeding season NAO index for most species (91.3%) (Table SM2). This was true for the majority of resident, partial migrant and fully migrant species (Table SM2)

The mean UK temperature remained stable between 1970 and 1994 in winter (September – March temperature trend = 0.008 ± 0.010, t = 0.54, P = 0.595) and during the breeding season (April – August temperature trend = 0.018 ± 0.014°C, t = 1.26, P = 0.22). It was also stable between 1995 and 2019 in the winter (September – March temperature trend = 0.009 ± 0.019, t = 0.46, P = 0.647) and the breeding season (April – August temperature trend = 0.014 ± 0.013°C, t = 1.12, P = 0.274) (Fig. SM1). However, there were higher mean temperatures from 1995 – 2019 than from 1970 – 1994 in winter (mean September - March temperatures = 12.5°C and 11.7°C respectively, t = 5.80, P < 0.001) and the breeding season (mean April - August temperatures = 6.8°C and 6.1°C respectively, t = 4.06, P < 0.001) (Fig. SM1).

**Table SM1** The percent of species with the lowest AIC value for each model with winter weather variables.

|  | Sept - March temperature | Sept - March NAO index | Nov - Feb temperature | Nov - Feb NAO index |
| --- | --- | --- | --- | --- |
| Percent species with best fit from weather variable | 80.4 % | 8.7 % | 2.2 % | 8.7 % |

**Table SM2** The percent of species with the lowest AIC value for each model with breeding season weather variables.

|  |  | UK breeding season temperature | Breeding season NAO index |
| --- | --- | --- | --- |
| Percent species with best fit from weather variable | **All species** | 91.3 % | 8.7 % |
|  | Resident | 92.9 % | 7.1 % |
|  | Partial migrant | 93.3 % | 6.7 % |
|  | Migrant | 88.2 % | 11.8 % |

**Figure SM1** Mean UK April – August temperature (solid lines and dark grey) and September – March temperature (dashed lines and light grey) with linear trend within time periods (early = 1970 – 1994; late = 1994 – 2019) and 95 % confidence intervals shown in grey.

*References*

Hollis, D., McCarthy, M., Kendon, M., Legg, T., & Simpson, I. (2019). HadUK‐Grid—A new UK dataset of gridded climate observations. Geoscience Data Journal, 6(2), 151-159.

Hurrell, J. W. (1995). Decadal trends in the North Atlantic Oscillation: Regional temperatures and precipitation. Science, 269(5224), 676-679.

Hurrell, J. W. & National Center for Atmospheric Research Staff (Eds). Last modified 24 Apr 2020. “The Climate Data Guide: Hurrell North Atlantic Oscillation (NAO) Index (station-based).” Retrieved from <https://climatedataguide.ucar.edu/climate-data/hurrell-north-atlantic-oscillation-nao-index-station-based> [accessed 22/03/2022].

Met Office (2018) HadUK-Grid gridded and regional average climate observations for the UK. Centre for Environmental Data Analysis. <https://catalogue.ceda.ac.uk/uuid/4dc8450d889a491>
